# Targeting rehabilitation to improve outcomes after total knee arthroplasty in patients at risk of poor outcomes: randomised controlled trial

**DOI:** 10.1136/bmj.m3576

**Published:** 2020-10-13

**Authors:** David F Hamilton, David J Beard, Karen L Barker, Gary J Macfarlane, Christopher E Tuck, Andrew Stoddart, Timothy Wilton, James D Hutchinson, Gordon D Murray, A Hamish R W Simpson, TRIO investigators, Caroline Austrie, Kim Brown, Matthew Carr, Ivan Brenkel, Tom Briggs, Katherine Dillow, Jamila Kassam, Ben Lankester, Emma McLoughlin, Helen Samuel, Jason Seaton, Kate Weatherly

**Affiliations:** 1 Department of Orthopaedics and Trauma, University of Edinburgh, Edinburgh EH16 4SB, UK; 2 Nuffield Department of Orthopaedics, Rheumatology and Musculoskeletal Sciences, University of Oxford, Oxford, UK; 3 Aberdeen Centre for Arthritis and Musculoskeletal Health (Epidemiology) Group, University of Aberdeen, Aberdeen, UK; 4 Usher Institute of Population Health, University of Edinburgh, Edinburgh, UK; 5 Department of Orthopaedics, Royal Derby Hospital, Derby, UK; 6 Department of Orthopaedic Surgery, University of Aberdeen, Aberdeen, UK

## Abstract

**Objective:**

To evaluate whether a progressive course of outpatient physiotherapy offers superior outcomes to a single physiotherapy review and home exercise based intervention when targeted at patients with a predicted poor outcome after total knee arthroplasty.

**Design:**

Parallel group randomised controlled trial.

**Setting:**

13 secondary and tertiary care centres in the UK providing postoperative physiotherapy.

**Participants:**

334 participants with knee osteoarthritis who were defined as at risk of a poor outcome after total knee arthroplasty, based on the Oxford knee score, at six weeks postoperatively. 163 were allocated to therapist led outpatient rehabilitation and 171 to a home exercise based protocol.

**Interventions:**

All participants were reviewed by a physiotherapist and commenced 18 sessions of rehabilitation over six weeks, either as therapist led outpatient rehabilitation (progressive goal oriented functional rehabilitation protocol, modified weekly in one-one contact sessions) or as physiotherapy review followed by a home exercise based regimen (without progressive input from a physiotherapist).

**Main outcome measures:**

Primary outcome was Oxford knee score at 52 weeks, with a 4 point difference between groups considered to be clinically meaningful. Secondary outcomes included additional patient reported outcome measures of pain and function at 14, 26, and 52 weeks post-surgery.

**Results:**

334 patients were randomised. Eight were lost to follow-up. Intervention compliance was more than 85%. The between group difference in Oxford knee score at 52 weeks was 1.91 (95% confidence interval −0.18 to 3.99) points, favouring the outpatient rehabilitation arm (P=0.07). When all time point data were analysed, the between group difference in Oxford knee score was a non-clinically meaningful 2.25 points (0.61 to 3.90, P=0.01). No between group differences were found for secondary outcomes of average pain (0.25 points, −0.78 to 0.28, P=0.36) or worst pain (0.22 points, −0.71 to 0.41, P=0.50) at 52 weeks or earlier time points, or of satisfaction with outcome (odds ratio 1.07, 95% confidence interval 0.71 to 1.62, P=0.75) or post-intervention function (4.64 seconds, 95% confidence interval −14.25 to 4.96, P=0.34).

**Conclusions:**

Outpatient therapist led rehabilitation was not superior to a single physiotherapist review and home exercise based regimen in patients at risk of poor outcomes after total knee arthroplasty. No clinically relevant differences were observed across primary or secondary outcome measures.

**Trials registration:**

Current Controlled Trials ISRCTN23357609 and ClinicalTrials.gov NCT01849445.

## Introduction

Total knee arthroplasty is a common procedure for end stage osteoarthritis of the knee, with more than 100 000 knee arthroplasties performed each year in the United Kingdom^1^ and more than 700 000 in the United States.^2^ Projections of future surgical volumes suggest further substantial increases.^3^ ^4^ ^5^ Although total knee arthroscopy is effective at reducing pain and improving physical function for most patients, around 20% report dissatisfaction with the postoperative outcome.^2^ ^6^

Physiotherapy is generally thought to be important in achieving optimal results after knee arthroplasty, yet the content of rehabilitation varies worldwide.^7^ Some countries such as the US and Australia use prolonged postoperative inpatient rehabilitation, although the effectiveness of this in improving outcomes (compared with outpatient based physiotherapy) has been questioned.^8^ In the UK, inpatient rehabilitation is of short duration, with patients typically discharged 3-5 days after arthroplasty. Under this model, physiotherapy during the brief inpatient stay is aimed at encouraging mobilisation and facilitating a safe discharge. Subsequent rehabilitation provision after discharge varies widely^9^ ^10^ and no definitive guidelines exist for rehabilitation after knee replacement. The generally held assumption that increased therapist contact enhances the rehabilitation has recently been challenged. Meta-analyses suggest that uniform postoperative physiotherapy for all patients after total knee arthroplasty compared with no treatment offers short term benefit but is not effective at improving patient outcomes at one year.^11^ ^12^ ^13^ Further recent meta-analysis of randomised trials suggests no difference in patient outcomes when rehabilitation consists of home or outpatient based interventions after total knee arthroplasty (with conclusions based on weak to moderate evidence).^14^ ^15^ However, as most patients report a good result after total knee arthroplasty, it might be that subgroups of patients could benefit from a targeted physiotherapy intervention. We determined if a six week programme of outpatient physiotherapy offers superior outcomes to a home exercise based regimen when targeted, in the early postoperative phase, at patients with predicted poor recovery and clinical outcomes after total knee arthroplasty.

## Methods

The TRIO (Targeted Rehabilitation to Improve Outcome) study was a multicentre parallel group randomised controlled trial evaluating the effect of different physiotherapy interventions targeted at patients with poor recovery six weeks after total knee arthroplasty. The study was carried out according to the published protocol.^16^

The study was registered before participant recruitment, which began in September 2013. The registration documents lacked detail of the planned secondary outcomes, which were detailed in the trial protocol document (published in the first months of recruitment). Because it is important to report as per the trial registration, both the secondary outcomes and the planned secondary outcomes that were not reported in the trial registration documents are provided separately in the results. As we planned to collect all data at the onset of recruitment, data on baseline outcomes are available for all trial participants.

### Recruitment and consent

Participants were recruited from 13 hospitals that provide total knee arthroplasty in the UK. Patients underwent routine surgical procedures at the study centres, which utilise local standard implants and techniques. All patients received immediate postoperative physiotherapy on the hospital wards, promoting mobility and knee range of motion and aimed at safe hospital discharge. Patients were screened for trial eligibility at routine clinical review six weeks after total knee arthroplasty. To be eligible for the study, patients had to have undergone a primary total knee arthroplasty for osteoarthritis and be at risk of a poor outcome (defined as an Oxford knee score of ≤26 points,^17^ ^18^ completed at that six week postoperative time point). The Oxford knee score measures pain and function after knee arthroplasty. The defined cut point of 26 points^17^ highlights those patients with most knee specific pain and dysfunction from simple activities, such as raising from a chair or mobilising a short distance. We excluded patients who were unwilling or unable to comply with the rehabilitation protocols, underwent arthroplasty purely for pain relief (ie, those with no expectation of mobilising postoperatively), required complex revision procedures, could not, or were unwilling to, attend their local outpatient department for rehabilitation, or had already received structured ongoing outpatient physiotherapy at six weeks post-surgery. Research nurses screened patients at the local sites and completed screening logs to record reasons for ineligibility.

### Randomisation and protection against bias

The local team carried out randomisation through a secure web based service supported by the Edinburgh Clinical Trials Unit. Randomisation was on a 1:1 basis, stratified by centre, with block allocation. As the intervention groups had clearly differing types and locations of physiotherapy, participants could not be masked to group assignment. The physiotherapists delivering the intervention could not be blinded to the intervention and thus were not involved in the study assessments beyond recording the timed-get-up-and-go test before and after the treatment intervention. Participants self-reported the primary outcome and secondary outcomes. To minimise bias, statistical analysis was carried out blinded to allocation.

### Interventions

All participants were reviewed by a physiotherapist two weeks after recruitment (eight weeks after total knee arthroplasty), at which point they were provided with rehabilitation advice and education about recovery, pain management, and pacing of activities. The participants then commenced 18 exercise sessions over six weeks and documented their exercise in a rehabilitation diary. On completion of the six week intervention all participants received a final session with the physiotherapist to review progress. The exercise diary was reviewed at the end of this study intervention visit and adherence was documented. After completion of the trial interventions at week 14, participants were advised to continue to progress their rehabilitation and exercise levels independently over the postoperative year. The published protocol includes full details of the trial interventions.^16^

#### Outpatient therapist led rehabilitation

The outpatient therapist led group undertook a progressive functional rehabilitation protocol (reviewed and modified weekly in a one-to-one contact session). The specific rehabilitation intervention for this study was based on best evidence for functional outcome.^19^ We used a goal led protocol that incorporated four categories: range of motion, strengthening, proprioception, and walking gait. Rehabilitation focused on agreed patient goals within these categories, with amendments over the six weeks as required. Although target goals were suggested for each category, the physiotherapists had flexibility in deciding how the goals could be achieved using local facilities and equipment, with a focus on exercise rehabilitation. Participants undertook a further two sessions of personalised rehabilitation each week at home. The physiotherapist directed this additional rehabilitation, with review and progression at the weekly contact session. To ensure standardisation of the study rehabilitation protocol and interventions, training sessions took place with the trial physiotherapists at arranged visits.

#### Physiotherapy review plus home exercise based regimen

The home exercise based group comprised a minimum standard-of-care intervention that reflects the provision of postoperative physiotherapy across the UK. After the initial physiotherapist review, participants were instructed to adhere to a self-directed home exercise based protocol, which focused on unloaded bending of the knee to promote range of motion and using the weight of the limb to strengthen the quadriceps muscle (with a stationary knee). Eighteen self-directed sessions were performed over the six weeks (three times weekly).

### Assessments

Study baseline assessment followed the clinical review six weeks post-surgery. Baseline data were collected before randomisation. We also collected outcome data at this point and then at 14, 26, and 52 weeks post-surgery by postal questionnaire. Reminder questionnaires were sent to non-responders after two weeks and followed-up by telephone if necessary. The physiotherapy teams collected additional physical performance data at weeks 8 and 14, before and after the six weeks of treatment intervention.

### Outcomes

#### Primary outcome

We chose the patient reported Oxford knee score as the primary outcome because it specifically measures the outcomes of knee arthroplasty^20^ ^21^ and is routinely used in the UK. Scores range from 0 (worst) to 48 (best), with 4 points indicating a minimum clinically important difference.^22^

#### Secondary outcomes

Global knee pain severity was assessed using an 11 point (0-10) visual analogue scale, where 0 represents no pain and 10 the worst possible pain, with 1.1 points representing the minimum important clinical difference.^23^ The validity and sensitivity of the visual analogue scale has been well documented.^24^ We performed separate assessments of worst pain and of perceived mean daily pain, as recommended in clinical trials of osteoarthritis.^25^

Participants were asked to rate their satisfaction with the operated knee on a 4 point Likert scale (very satisfied, satisfied, unsure, or dissatisfied). Further single item questions inquired as to “how well the surgery relieved pain in the affected joint,” “how well the surgery increased ability to perform regular activities,” and ‘how well the surgery increased the ability to perform heavy work or sport activities” on the same 4 point Likert scale.

The timed-get-up-and-go test was performed before and after the intervention. This is a simple test used to assess an individual’s mobility, requiring both static and dynamic balance. It is the time taken to rise from a chair, walk three metres, turn around, walk back to the chair, and sit down. The performance of this test has been found to decrease substantially with mobility impairments.^26^ ^27^

### Statistical analysis

The primary endpoint was the 52 week Oxford knee score. During the development of the trial, the accepted minimum clinically important difference of this score was 3 points.^21^ Based on an α of 0.05 and an assumed standard deviation of 9.2, we determined that 300 patients would be required to detect a minimum clinically important difference of 3 points in Oxford knee score at 52 weeks with an 80% power and 400 patients for a 90% power.^16^

We performed an intention-to-treat analysis, incorporating all randomised participants. The primary outcome was evaluated using analysis of covariance, with baseline Oxford knee score and study centre as covariates in a fixed effects model. For this we did not impute missing data. The initial analysis was confirmed using multiple imputation to explore the impact of missing data for the 52 week score. Post hoc analyses of the primary outcome included a longitudinal analysis of covariance including all time point data (baseline and 14, 26, and 52 weeks) to evaluate trajectory of recovery. We used the same analysis of covariance methods to evaluate secondary outcomes at the various postoperative time points.

We evaluated the analysis of covariance model of the timed-get-up-and go test with data recorded before and after the treatment intervention. Patient satisfaction was evaluated using ordinal regression, with baseline (sixth postoperative week) Oxford knee score, study centre, and the allocated intervention included as covariates.

Sensitivity analyses were performed on the per protocol sample using the same process. We defined adherence to the allocated intervention as no additional physiotherapy in the home exercise based group and as attendance of at least four of the six hospital based sessions in the therapist led outpatient intervention group.

The statistician conducting the analyses was blinded to group allocation. SAS (v9.2) was used for statistical analyses (SAS Institute), with two sided tests at a level of 0.05 considered to be significant.

#### Protocol deviations

The trial was originally powered to detect a 3 point difference in the Oxford knee score between intervention arms; the accepted minimum clinically important difference. Subsequent research (during trial recruitment) suggested that a 4 point difference would be required to show an observable or meaningful difference in patient outcome.^22^ As such we elected to interpret our trial with the newly defined 4 point difference. The independent trial steering committee approved this change. At the time of this change we had recruited 334 patients, and as the 4 point difference allowed in excess of 90% power to detect a between group difference, we closed recruitment. The health economic analysis for this trial will be reported separately.

### Patient and public involvement

The study was developed in response to a survey of patients, which highlighted ambiguity and concern as to the correct amount of postoperative physiotherapy that should be undertaken after knee arthroplasty. The trial was developed at a workshop hosted by Arthritis Research UK (now Versus Arthritis) osteoarthritis clinical study group, which included surgical, physiotherapeutic, and patient representatives. The study steering group benefitted from a patient representative.

## Results

**Fig 1:**
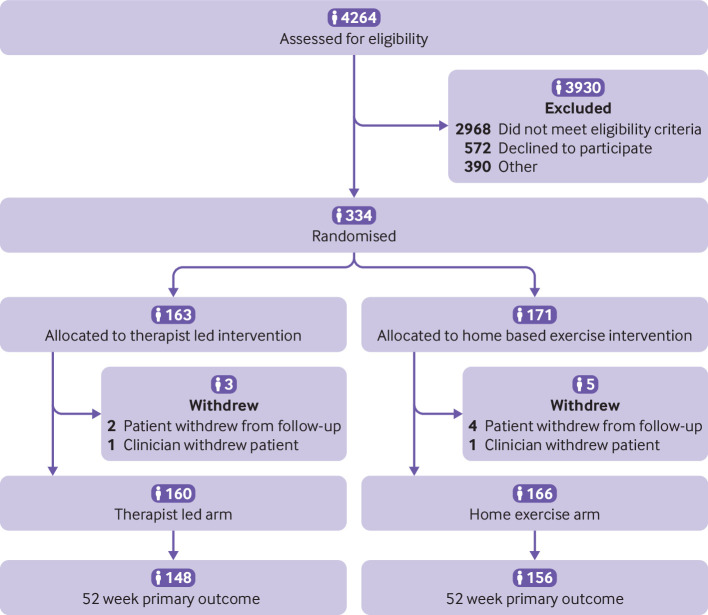
Flow of participants through the study

Overall, 4264 patients were screened for eligibility of whom 334 were randomised. Most of the screened patients (n=2968, 69.6%) were not eligible for the study, enabling the rehabilitation intervention to be targeted to those defined as at risk of poor outcomes. Figure 1 outlines the reasons for non-recruitment. In addition to the 2968 ineligible patients, 572 declined to participate in the study. This number includes those who did not have equipoise to be randomised (ie, those who preferred a defined local rehabilitation), were unable to attend outpatient rehabilitation, and had no interest in taking part. In total, 390 patients were coded as other, which incorporated those taking part in another physiotherapy trial and problems with site logistics such as unavailability of research staff to recruit. The three patients with data missing on why they did not enrol are coded as other in the study flowchart.

Eight of the 334 randomised participants were lost to follow-up. Six participants withdrew from follow-up (n=2 (1.6%) therapist led group, n=4 (2.3%) home exercise based group). A further two participants (one (0.6%) in each trial arm) were withdrawn from the study by their clinician.

Overall mean age of the randomised cohort was 67.5 (SD 9.46) years, 61.4% (n=205) were women, and overall mean body mass index was 31.34 (SD 5.67). The supplementary file lists the personal characteristics of screened and recruited patients. Table 1 summarises the baseline characteristics of the participants by study arms. Preoperative data for the primary outcome were available for a subset of centres; these data confirmed no difference in the average preoperative presentation between groups (see supplementary file).

**Table 1:** Baseline characteristics of participants assigned to outpatient physiotherapist led or home exercise based intervention after total knee arthroplasty. Values are numbers (percentages) unless stated otherwise

Characteristics	Overall (n=334)	Therapist led (n=163)	Home exercise (n=171)
Women	205 (61.4)	97 (59.3)	108 (62.2)
Mean (SD) age (years)	67.5 (9.46)	66.8 (9.46)	68.2 (9.44)
Mean (SD) body mass index	31.34 (5.76)	31.19 (5.30)	31.50 (6.18)
Comorbidity*:			
Heart disease	58 (17.4)	34 (20.9)	24 (14.0)
Hypertension	154 (46.1)	73 (44.8)	81 (47.4)
Lung disease	45 (13.5)	20 (12.3)	25 (14.6)
Stroke	11 (3.3)	6 (3.7)	5 (2.9)
Kidney disease	2 (0.6)	1 (0.6)	1 (0.6)
Liver disease	2 (0.6)	0 (0)	2 (1.2)
Stomach ulcer	23 (6.9)	14 (8.6)	9 (5.3)
Anaemia	13 (3.9)	5 (3.1)	8 (4.7)
Depression	39 (11.7)	24 (14.7)	15 (8.8)
Low back pain	82 (24.6)	42 (25.8)	40 (23.4)
Other pain	147 (44)	74 (45.4)	73 (42.7)
Mean (SD) Oxford knee score	20.24 (4.78)	20.40 (4.82)	20.08 (4.74)
Mean (SD) timed-get-up-and-go	21.66 (63.13)	18.85 (44.8)	24.33 (80.6)
Mean (SD) worst pain	6.15 (1.84)	6.06 (1.88)	6.23 (1.81)
Mean (SD) average pain	4.82 (1.77)	4.71 (1.76)	4.93 (1.77)

* Patients who reported drug controlled comorbidity.

Of the 334 randomised participants, 163 were allocated to therapist led outpatient rehabilitation and 171 to home exercise based rehabilitation. Compliance with treatment was 85.3% in the therapist led group and 97.7% in the home exercise group. The primary outcome was recorded for 148 (91%) participants in the therapist led group and 156 (91%) in the home exercise based group.

### Primary outcome

In the intention-to-treat analysis, the adjusted mean between group difference in Oxford knee score at one year was 1.91 (95% confidence interval −0.18 to 3.99) points favouring the therapist led group (P=0.07). The result was similar in the per protocol analysis (2.02, −0.15 to 4.18) points, P=0.07).

### Secondary outcomes

Both intervention arms showed an improvement in Oxford knee score (between baseline and 52 weeks) in excess of the 4 point minimum clinically important difference. When all time point data were analysed, the between group difference was 2.25 points (95% confidence interval 0.61 to 3.90, P=0.01).

Postoperative differences in the timed-get-up-and-go test were not significant when accounting for baseline scores (table 2 and supplementary file). Overall satisfaction did not differ between the groups (odds ratio 1.07, 95% confidence interval 0.71 to 1.62); however, enhanced satisfaction with pain relief (1.66, 1.10 to 2.52), ability to perform daily functional tasks (1.66, 1.09 to 2.51), and ability to perform heavy functional tasks (1.57, 1.02 to 2.42) was reported in the therapist led group (table 3).

**Table 2:** Outcomes at 14, 26, and 52 weeks after total knee arthroplasty

Outcomes	No of participants			Mean (SD)		Mean (95% CI) between group difference*	P for difference
	Therapist led	Home exercise		Therapist led	Home exercise		
**Primary outcome**							
Oxford knee score at 52 weeks:							
Intention to treat	148	156		33.55 (10.06)	31.57 (9.68)	1.91 (−0.18 to 3.99)	0.07
Per protocol	148	151		33.76 (9.90)	31.56 (9.75)	2.02 (−0.15 to 4.18)	0.07
**Secondary outcomes**							
Oxford knee score:							
14 weeks	154	151		31.80 (7.64)	30.20 (8.13)	1.60 (0.05 to 3.16)	0.04
26 weeks	150	151		32.12 (8.81)	30.34 (8.75)	1.70 (−0.11 to 3.51)	0.07
Timed-get-up-and-go (seconds) at 14 weeks	143	143		14.65 (38.0)	22.5 (77.2)	4.64 (−14.25 to 4.96)	0.34
**Outcomes not listed in trial registration documents**							
Worst pain:							
14 weeks	154	150		3.97 (2.46)	4.44 (2.41)	0.46 (−0.98 to 0.07)	0.09
26 weeks	148	148		3.80 (2.61)	4.22 (2.55)	0.32 (−0.86 to 0.22)	0.24
52 weeks	147	156		3.36 (2.92)	3.64 (2.80)	0.22 (−0.71 to 0.41)	0.50
Average pain:							
14 weeks	154	150		2.87 (2.09)	3.21 (2.06)	0.29 (−0.71 to 0.14)	0.19
26 weeks	147	147		3.02 (2.21)	3.45 (2.25)	0.26 (−0.72 to 0.19)	0.25
52 weeks	147	154		2.72 (2.52)	3.09 (2.51)	0.25 (−0.78 to 0.28)	0.36

* Adjusted for baseline score and study centre.

**Table 3:** Patient satisfaction at one year after total knee arthroplasty, by treatment group. Values are numbers (percentages) unless stated otherwise

Satisfaction question	Positive response		Odds ratio (95% CI) for difference*	P for difference
	Therapist led	Home exercises		
How satisfied are you with your operated knee?	95 (58.3)	90 (52.6)	1.07 (0.71 to 1.62)	0.75
How well did the surgery:				
relieve the pain in your affected joint?	108 (66.3)	94 (55.0)	1.66 (1.10 to 2.52)	0.02
increase your ability to perform regular activities?	89 (54.6)	75 (43.9)	1.66 (1.09 to 2.51)	0.02
allow you to perform heavy work or sports activities?	46 (28.3)	43 (25.1)	1.57 (1.02 to 2.42)	0.04

Denominators for analysis were all randomised participants and not just those satisfied.

* Adjusted for baseline Oxford knee score, study centre, and allocated intervention.

### Planned secondary outcomes not reported in trial registration documents

Small, non-significant reductions in worst and average pain scores were observed favouring the therapist led group. These reductions were substantially below the minimum clinically important difference of 1.1 points for this metric (table 2).

## Discussion

In this study, no statistical or clinically meaningful differences were found for patient reported pain or functional outcomes in those at risk of poor outcomes after total knee arthroplasty between outpatient physiotherapy and a single physiotherapy review and home exercise based regimen. Overall satisfaction with the operated knee at one year did not differ between the groups; however, those who received the therapist led intervention reported enhanced satisfaction with pain relief and the ability to undertake physical activities.

### Strengths and limitations of this study

Specific strengths of this study include the comparatively large sample size and low losses to-follow-up, which along with good treatment adherence ensured high study power to detect a between group difference. One limitation is that we did not include a no treatment group for comparison. Although there is debate about the effectiveness of physiotherapy after total knee arthroplasty, and ambiguity about the best delivery method, it remains an accepted component of the treatment pathway for knee arthroplasty in the UK. As such it was considered unethical to include a control group of high risk participants who would not receive physiotherapy. As is the case with most physiotherapy trials, it was not feasible to blind patients or therapists to treatment allocation. To ensure generalisability and limit excess treatment costs, we compared physiotherapy interventions that are deliverable within the constraints of national health service resources. Standard commissioning in the NHS limits physiotherapy provision to about six sessions, therefore we developed our protocols to reflect this provision. A more intensive rehabilitation intervention might have had a beneficial effect; however, studies utilising more comprehensive and lengthy rehabilitation protocols have also found no benefit.^10^ ^11^ ^12^ ^13^ ^14^ ^15^ Responder bias was possible as the outcomes were primarily patient reported, although such bias would probably favour the therapist led arm as physiotherapy contact was greater, and the generalisability to other healthcare settings outside the NHS is assumed but undefined.

### Implications for patients and policy

Consensus as to the best way to deliver rehabilitation after total knee arthroplasty is lacking. Specific protocols are strongly entrenched at individual physiotherapy departments despite the wider efficacy of postoperative physiotherapy being poorly established.^28^ This uncertainty about effectiveness of physiotherapy makes it difficult for patients, commissioning organisations, and healthcare providers to determine the best way to deliver physiotherapy after total knee arthroplasty and the correct level and mechanism of funding for such services.

It is increasingly evident that patient outcomes one year after total knee arthroplasty are not influenced by the location or type of postoperative rehabilitation when applied to all patients.^10^ ^11^ ^12^ ^13^ ^14^ ^15^ We targeted physiotherapy interventions specifically at patients considered to be at most risk of poor outcomes. Predicting preoperatively which patients will do well or poorly after total knee arthroplasty has proved challenging despite the large numbers of patients who undergo this procedure annually. Personal factors relating to patient heath and symptoms are a useful general guide, although these preoperative indicators do not predict those who will benefit from physiotherapy postoperatively.^9^ We therefore recruited poorly performing patients on the basis of actual functional status at the time of early (six week) postoperative clinical review, targeting our interventions to those presenting with higher than normal levels of knee specific pain, finding it difficult to perform simple tasks of daily living, and most vulnerable to experiencing poor clinical outcomes.

Why some patients experience poorer recovery after knee arthroplasty is not well understood and might suggest that physiotherapy exerts a marginal influence on the underlying causes of poor post-surgery outcomes—for example, functional limitations that might relate to implant positioning, and intraoperative factors that might not be modifiable with postoperative physiotherapy (irrespective of content). Other patient factors such as comorbid conditions, local muscle deficits, and attitudes towards health or catastrophising behaviours might also limit responsiveness to postoperative rehabilitation.

Although we selectively recruited only those considered at risk of poor outcomes, the mean (trial cohort) Oxford Knee Score at one year was 4-6-points below the wider UK average of 36 points^29^ for all patients after knee arthroplasty, suggesting that even those who show poorer recovery in the early postoperative phase can achieve reasonable outcomes by one year with a rehabilitation intervention and that the method of delivery and content of the physiotherapy received might be comparatively unimportant. It could be that simply making patients aware that they are at risk of poor outcomes and providing hands-off rehabilitation through physiotherapist review and a home exercise based regimen are enough to optimise outcomes; in this study we found no benefit of providing an alternative more comprehensive physiotherapist facilitated outpatient rehabilitation.

### Conclusions

In this randomised trial targeting physiotherapy to patients at risk of poor outcomes after total knee arthroplasty, we observed no meaningful differences in patient outcome or overall satisfaction with outcome at one year as a result of patients undertaking outpatient physiotherapy compared with a single physiotherapy review and home exercise based regimen. Although targeting rehabilitation interventions to at risk patients is a feasible delivery method, the content of the rehabilitation seems to have minimal influence on patient outcomes.

**What is already known on this topic**

Despite physiotherapy after total knee arthroplasty being considered important for optimal results, rehabilitation varies worldwide
Differing postoperative rehabilitation approaches have not been shown to deliver meaningful differences in patient outcomes after surgery
This finding is based on physiotherapy applied to all patients, so it is unclear whether people at risk of poor recovery would benefit from more intensive rehabilitation

**What this study adds**

This study suggests that targeting postoperative physiotherapy to those most in need is a feasible delivery method, but that the actual content of the rehabilitation seems to have minimal influence on patient outcomes
That there was no additional benefit of physical outpatient rehabilitation compared with single physiotherapist review and home exercise based could have implications for healthcare delivery and resource planning around this high volume procedure

## Web extra

Extra material supplied by authors

## Notes

Ethical approval: The trial protocol was approved by the South East Scotland Research Ethics Committee 01 (13/SS/0051) and local research and development departments at participating sites.

Data sharing: Anonymised patient level data can be made available on reasonable request and after approval by a review panel. Requests should be made to ECTUdatashare@ed.ac.uk.

The lead author affirms that the manuscript is an honest, accurate, and transparent account of the study being reported; that no important aspects of the study have been omitted; and that any discrepancies from the study as originally planned (and, if relevant, registered) have been explained.

Dissemination to participants and related patient and public communities: We intend to disseminate the results of this study to the participants through Versus Arthritis and the local hospital physiotherapy departments.

Provenance and peer review: Not commissioned; externally peer reviewed.
