# Supplementary material for: Targeting rehabilitation to improve outcomes after total knee arthroplasty in patients at risk of poor outcomes: randomised controlled trial
Source: BMJ. 2020 Oct 13;371:m3576. doi: 10.1136/bmj.m3576 (PMC7551789; doi:10.1136/bmj.m3576)
Supplement: Supplementary file 1 — Supplementary information: additional tables [file hamd053756.ww.pdf]

Supplemental material

Characteristics of randomised and not-recruited participants

	**Overall** (n=4264)	**Recruited** (n=334)	**not-recruited** (n=3930)
Sex, n (%) female	2399 (56.3)	205 (61.4)	2194 (55.8)
Age, mean (SD)	69.54 (9.55)	67.52 (9.46)	69.72 (9.54)
Baseline OKS, mean (SD)	28.39 (8.84)	20.24 (4.78)	29.21 (8.33)

Preoperative Oxford Knee Score

	**Overall** (n=153)	**Therapist-led** (n=77)	**Home-exercise** (n=76)
Preoperative OKS, mean (SD)*	17.06 (7.18)	17.86 (7.39)	16.25 (6.91)

*linked data retrospectively available for patients undergoing TKA in NHS Lothian and NHS Fife

Timed-get-up-and-go

	**Overall** (n=286)	**Therapist-led** (n=143)	**Home-exercise** (n=143)
Timed- up-and-go, mean (SD)	18.58 (60.84)	14.65 (38.00)	22.50 (77.16)

Adjusted mean difference* 4.65 (95%CI -14.3-4.7) p=0.34

*Adjusting factors: baseline (8-week) timed-get-up-and-go and study centre
